# Gangliosides Contribute to Vascular Insulin Resistance

**DOI:** 10.3390/ijms20081819

**Published:** 2019-04-12

**Authors:** Norihiko Sasaki, Yoko Itakura, Masashi Toyoda

**Affiliations:** Department of Geriatric Medicine (Vascular Medicine), Tokyo Metropolitan Institute of Gerontology, Sakaecho 35-2, Itabashi-ku, Tokyo 173-0015, Japan; yitakura@tmig.or.jp

**Keywords:** endothelial cell, aging, senescence, inflammation, GM1 ganglioside, vascular insulin resistance

## Abstract

Insulin in physiological concentrations is important to maintain vascular function. Moreover, vascular insulin resistance contributes to vascular impairment. In the elderly, other factors including hypertension, dyslipidemia, and chronic inflammation amplify senescence of vascular endothelial and smooth muscle cells. In turn, senescence increases the risk for vascular-related diseases such as arteriosclerosis, diabetes, and Alzheimer’s disease. Recently, it was found that GM1 ganglioside, one of the glycolipids localized on the cell membrane, mediates vascular insulin resistance by promoting senescence and/or inflammatory stimulation. First, it was shown that increased GM1 levels associated with aging/senescence contribute to insulin resistance in human aortic endothelial cells (HAECs). Second, the expression levels of gangliosides were monitored in HAECs treated with different concentrations of tumor necrosis factor-alpha (TNFα) for different time intervals to mimic in vivo acute or chronic inflammatory conditions. Third, the levels of insulin signaling-related molecules were monitored in HAECs after TNFα treatment with or without inhibitors of ganglioside synthesis. In this review, we summarize the molecular mechanisms of insulin resistance in aged/senescent and TNFα-stimulated endothelial cells mediated by gangliosides and highlight the possible roles of gangliosides in vascular insulin resistance-related diseases.

## 1. Introduction

Insulin resistance is typically defined as the reduced ability of insulin to induce glucose uptake by classic targets such as fat, liver, and skeletal muscle, leading to a common manifestation of obesity and a prelude to type 2 diabetes. However, insulin receptors (IRs) are not exclusively restricted to those tissues and are present in several cell types including vascular cells. Vascular endothelial cells (ECs), which constitute the endothelium of blood vessels, play pivotal roles in vascular homeostasis. Excessive activation or dysfunction of ECs leads to the development of vascular-related diseases, such as restenosis and arteriosclerosis [1]. Insulin signaling in ECs contributes to the regulation of important EC functions and to the maintenance of vascular integrity. Nitric oxide (NO) production upon endothelial NO synthase (eNOS) activation through insulin signaling is known to maintain endothelial barrier integrity, which is important to avoid accumulation and retention of atherogenic apolipoprotein B-containing lipoproteins in the subendothelial space [2]. NO production also suppresses EC surface expression of adhesion molecules, inhibiting the accumulation of monocytes and monocyte-derived macrophages in the intimal region, and thus attenuating the induction and development of atherosclerosis [3,4,5,6]. It has been demonstrated that insulin resistance in ECs causes skeletal muscle insulin resistance due to reduced insulin-induced capillary recruitment and insulin delivery, leading to diabetes [7]. Vascular insulin resistance is therefore considered to play an important role in the pathogenesis of vascular and vascular-related diseases, including type 2 diabetes [6,7,8]. This review summarizes the possible roles of gangliosides in vascular insulin resistance and highlights future research directions in vascular insulin resistance-related diseases.

## 2. Regulation of Extrinsic Insulin Signaling Mediated by Gangliosides

**Figure 1:**
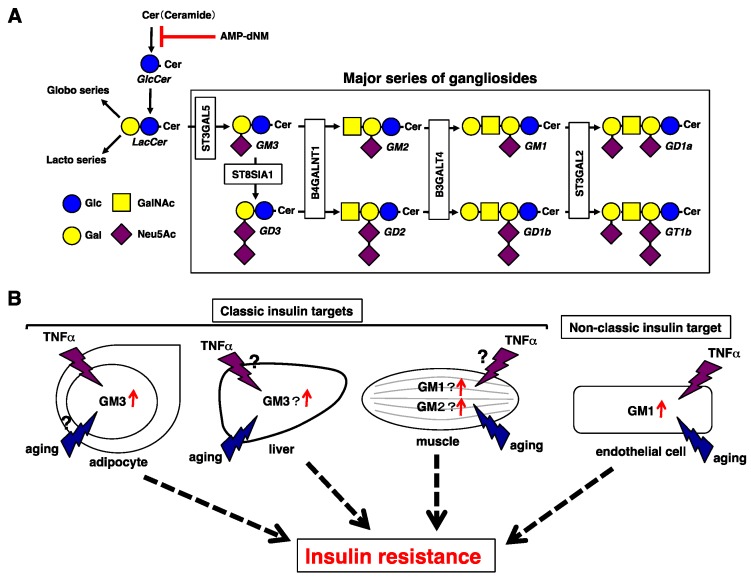
Insulin resistance mediated by gangliosides. (**A**) Pathways of glycolipids and glycosyltransferases contributing to each ganglioside synthetic pathway. Pathways of the major gangliosides are shown within the rectangle. AMP-dNM is a specific inhibitor of glucosylceramide synthase. Symbols are according to Reference [22]. Glc, Glucose; Gal, Galactose; GalNAc, *N*-acetylgalactosamine; Neu5Ac, *N*-acetylneuraminic acid. (**B**) Gangliosides involved in insulin resistance in classic or non-classic sites. In adipocytes, increased GM3 levels upon TNFα stimulation contribute to insulin resistance in pathological conditions such as obesity, but their contribution to aging is unknown. In the liver, GM3 contributes to insulin resistance possibly due to a reduction in *NEU3* that occurs with aging. In the muscle, GM1 and GM2 contribute to insulin resistance, and GM3 contributes to insulin resistance due to a reduction in *NEU3* that occurs with aging. In both the liver and the muscle, the effect of TNFα stimulation is unknown. In ECs, simultaneous aging and TNFα stimulation contribute to upregulation of GM1, leading to insulin resistance.

Glycosphingolipids, which are composed of sugar chains attached to the sphingolipid ceramide, have been used as cell surface markers and are known to regulate several biological functions [9,10,11]. Among glycosphingolipids, gangliosides, which are molecules composed of glycosphingolipids with one or more sialic acids linked (Figure 1A), are well-known key components of lipid rafts [12]. Changes in ganglioside levels are known to affect the expression of raft-associated proteins on the cell surface and lead to reduced membrane fluidity, resulting in cellular dysfunctions, such as impaired signal transduction [13,14,15]. It has been demonstrated that gangliosides contribute to regulation of insulin signaling and that changes in the composition of cell surface gangliosides lead to cellular responses associated with physio-pathological conditions. Increased levels of GM3 in 3T3-L1 adipocytes upon tumor necrosis factor-alpha (TNFα) stimulations were found to induce insulin resistance in proinflammatory conditions such as obesity [16]. Furthermore, in transgenic insulin-resistant mice, overexpression of *NEU3*, which is a plasma membrane-associated sialidase modulating ganglioside content by removing sialic acid, induced an increase in the levels of GM1 and GM2 mainly in muscles, indicating that GM1 and GM2 contribute to insulin resistance in muscles [17]. Another report has shown that reduction of GM3 in the liver via hepatic *NEU3* overexpression improved insulin sensitivity and glucose tolerance in mice, indicating that GM3 contributes to insulin resistance in the liver [18]. In addition, *NEU3* downregulation in the liver and muscle of aged mice led to insulin resistance, due to an increase in GM3 levels [19]. Thus, gangliosides attenuate insulin action on classical targets. Recently, it has been demonstrated that increased GM1 levels associated with aging/senescence and inflammatory conditions contribute to insulin resistance in non-classical targets cells, such as ECs [20,21]. Therefore, gangliosides play important roles in insulin resistance and the concentrations and types of gangliosides associated to insulin resistance differ among cell types and tissues (Figure 1B).

## 3. Vascular Insulin Resistance Mediated by Gangliosides in Aging/Senescence

Aged/senescent cells including ECs play important roles in vascular disease [23,24]. Blood vessels are present in the entire body, and aging of vascular cells is thought to affect systemic diseases as well. Therefore, elucidating the molecular mechanisms underlying aging/senescence-related diseases is important for lowering the risk of vascular disease and extending healthy life expectancy. It is now known that aging/senescence causes EC dysfunctions such as reduced NO production and elevated inflammation [25]. It was thus speculated that aging/senescence leads to insulin resistance in ECs, but the molecular mechanisms underlying this process were unclear.

### 3.1. Expression Levels of Gangliosides in Senescent ECs

To identify cell surface gangliosides involved in the senescence of ECs, ganglioside expression was compared between non-senescent and senescent human aortic endothelial cells (HAECs). Senescence was induced by long-term culture (replicative senescence) or H_2_O_2_ stress (stress-induced premature senescence). Senescent HAECs exhibited an enlarged and flattened morphology, a lowered proliferative capacity, and an increased amount of senescence-associated (SA)-β-gal-positive cells. Fluorescence-activated cell sorting (FACS) analysis of gangliosides revealed that GM1 and GD1a are mainly present on HAECs and that the levels of GM1 are significantly increased on senescent compared with non-senescent HAECs. Thus, it was shown that GM1 levels are increased on the surface of HAECs undergoing both replicative and premature senescence [20].

### 3.2. Regulation of Insulin Signaling Mediated by Gangliosides in Senescent ECs

Whether or not vascular insulin resistance occurs in senescent ECs upon increased GM1 levels has been the subject of recent research efforts. The insulin signaling cascade activates protein kinase B (Akt), which in turn phosphorylates and activates eNOS in ECs [26]. In senescent HAECs, insulin signaling is impaired and increased levels of GM1 contribute to insulin resistance [20]. In adipocytes, interaction between GM3 and IR is required for insulin resistance. This interaction is mediated by the sialic acid of GM3 [27]. Immunocytochemical and immunoprecipitation analyses clarified the interaction between GM1 and IR in senescent HAECs [20]. Two possible mechanisms of GM1-mediated insulin resistance in senescent ECs have been proposed. The first one is based on an association between the GM1 carbohydrate moiety and an epitope in the extracellular domain of IR, which leads to the exclusion of IR from caveolae, as it was recently found for GM3 and IR [27]. The second proposed mechanism suggests that increased levels of GM1 affect the catalytic activity of IR and impair signal transduction [17].

### 3.3. Expression Levels of Gangliosides in Aged ECs

An interesting possibility to test was whether aging also contributes to increased GM1 levels in ECs resulting in insulin resistance. ECs derived from individuals of different ages are used as in vitro models of human aging, as these cells show proliferative properties and a number of passages to senescence correlating with the age of origin. As ECs reflect individual age, they can be used as in vitro vascular aging models. The levels of GM1 in senescent HAECs, which were derived from an 81-year-old individual, were approximately six times higher than those of HAECs derived from younger subjects even at early passage. The high abundance of GM1 in senescent HAECs contributed to their insulin resistance [20]. This result suggested that an increase in the levels of GM1 occurs with aging and contributes to vascular insulin resistance in older people. In aged mice, it is known that downregulation of *NEU3* contributes to an increase in GM3 levels in the liver and muscle [19]. On the other hand, in senescent ECs, it was suggested that *B4GALNT1*, rather than *NEU3*, is involved in the observed increase in GM1 levels [20]. Therefore, contributors of ganglioside synthesis differ among cell types and tissues during aging.

## 4. Vascular Insulin Resistance Mediated by Gangliosides in Inflammatory Conditions

Inflammatory mediators have the potential to induce vascular insulin resistance [28]. It has been reported that serum levels of the pro-inflammatory cytokine TNFα are increased in obese and elderly people and correlate with a high prevalence of vascular diseases [29,30]. In high-fat diet-fed mice, phosphatase and tensin homologues upregulated by TNFα induce vascular insulin resistance [31]. Furthermore, it is known that production of TNFα by hypertrophic and senescent adipocytes induces insulin resistance in ECs [7,32]. Senescent ECs also produce TNFα, which probably has an autocrine/paracrine action [33]. Therefore, TNFα potentially contributes to vascular insulin resistance in elderly obese and normal-weight subjects.

### 4.1. Basics of TNFα Signaling

TNFα is a pleiotropic cytokine that induces signal transduction through homotrimeric TNF receptor 1 (TNFR1) and TNF receptor 2 (TNFR2) [34]. TNFR1 is expressed ubiquitously, whereas expression of TNFR2 is restricted to specific cell types, such as immune cells and ECs [35]. TNFα binding to TNFR1 recruits the adaptor molecule TNFR1-associated death domain protein, leading to activation of signaling molecules including nuclear factor κB (NFκB) and mitogen-activated protein kinases (MAPKs). TNFR1 signaling triggers several biological processes, such as induction of inflammation, tissue degeneration, cell survival, and host defense. On the other hand, TNFR2 signaling is induced primarily by cell–cell interactions via transmembrane TNF. TNFR2 recruits TNFR-associated factor 2, leading to the downstream activation of NFκB, MAPKs, and Akt. TNFR2 signaling contributes to homeostatic processes, such as tissue regeneration, cell proliferation, and cell survival. In human ECs, TNFR2 signaling induces autocrine interferon-β signaling followed by monocyte recruitment [36].

### 4.2. Regulation of Insulin Signaling Mediated by Gangliosides in Acute or Chronic Vascular Inflammatory Models

Inflammation has a detrimental effect on the function of vasculature when the mechanisms regulating the intensity and duration of an acute inflammatory response become compromised. For example, an excessive inflammatory response during sepsis leads to organ failure and death due to systemic and profound increases in the permeability of ECs, and chronic vascular inflammation leads to type 2 diabetes and progression of atherosclerosis [37,38,39]. However, the precise mechanisms of vascular insulin resistance induction via chronic inflammation have not been clarified. Since these mechanisms are highly complex, recapitulation of the in vivo situation is currently difficult. For this reason, in vitro experiments mimicking in vivo acute or chronic vascular inflammatory conditions in ECs have been designed. So far, several studies have investigated ECs treated with different concentrations of TNFα for different time intervals. For example, one report demonstrated that short-term (6 h) 2 ng/mL TNFα exposure upregulates intercellular adhesion molecule-1 in human umbilical vein ECs (HUVECs), leading to increased adhesion of monocytes to ECs [40]. Another report has shown that high concentrations (10 ng/mL) of TNFα induce morphological changes and enhance permeability within 24 h in an immortalized human cerebral endothelial cell line [41]. In addition, it has been recently reported that long-term (6 days) exposure to high concentrations (10 ng/mL) of TNFα induces premature senescence in HUVECs via reactive oxygen species production [42]. Therefore, the effect of TNFα varies depending on exposure time and concentration.

In 3T3-L1 adipocytes, low concentrations (1.7 ng/mL) of TNFα are known to upregulate GM3 [43]. On the other hand, it was unknown whether and what kinds of gangliosides are affected in ECs upon TNFα stimulation. A recent report has shown by FACS analysis of gangliosides in HAECs after short-term stimulation with several concentrations of TNFα (0.1 ng/mL–10 ng/mL) that GM1 expression on the cell surface increases in a concentration-dependent manner (with TNFα concentrations above 1 ng/mL) [20]. Thus, with regard to ganglioside expression, ECs seem to be more sensitive to TNFα than adipocytes. Among the other three main gangliosides (GM3, GM2, GD1a), GM3 and GM2 were undetectable, whereas GD1a levels were decreased in 1 ng/mL TNFα-treated HAECs. Thus, expression of gangliosides upon TNFα stimulation is different between adipocytes and ECs.

As an in vitro model of acute or chronic inflammation, we examined in our previous report the effect of short- (acute, 3 days) or long-term (chronic, 7 days) exposure to different concentrations of TNFα on insulin signaling in HAECs [21]. In short-term low-concentration (1 ng/mL) TNFα-treated HAECs, insulin signaling was impaired without any effect on expression of insulin signaling molecules. In contrast, short-term high-concentration (10 ng/mL) TNFα induced a reduction in insulin signaling and eNOS activation together with downregulation of insulin signaling molecules (IR substrate 2 and eNOS). These results suggest that in acute inflammatory conditions even low concentrations of TNFα can induce vascular insulin resistance due to increased cell surface expression of GM1. In long-term low-concentration (1 ng/mL) TNFα-treated HAECs, insulin signaling was impaired and eNOS was downregulated due to increased GM1 expression. In contrast, long-term high-concentration (10 ng/mL) TNFα showed the potential to induce cellular senescence [42]. In fact, in chronic inflammatory conditions, even low concentrations of TNFα induce downregulation of eNOS via increased GM1 levels. This suggests that activation of eNOS by insulin but also other factors, such as vascular endothelial growth factor [44], may be impaired, presumably leading to serious vascular diseases. At the moment, beraprost sodium is available as a potential therapeutic for vascular insulin resistance. The drug acts by upregulating eNOS levels [7]. In addition, anti-TNF therapy is used for chronic inflammatory diseases such as rheumatoid arthritis [45]. However, therapeutic strategies for vascular insulin resistance still need further development. The timing and the degree of vascular insulin resistance induction may be dependent on exposure time and/or concentration of TNFα. Therefore, not only therapies targeting eNOS and/or TNFα, but also extracellular therapeutic strategies targeting GM1 may be useful for prevention and cure of vascular insulin resistance, although further studies are required to evaluate the effects of GM1 systemic targeting.

### 4.3. Regulation of Insulin Signaling Mediated by Gangliosides in Homeostatic Models after Inflammatory Stimulation

Inflammation is a protective response that restores physiological functions when homeostatic systems are not sufficient. However, in states of homeostasis restoration, inflammation may enforce and propagate detrimental changes of homeostatic set point resulting in chronic pathological states [46]. For example, inflammation maintains and even worsens insulin resistance in obese patients [47]. In blood vessels, a prolonged inflammatory response affects detrimentally the function of vasculature, therefore appropriate control of inflammatory responses is essential [48,49]. A prolonged duration of vascular insulin resistance via increased GM1 expression may thus have a detrimental effect. In order to investigate homeostasis in TNFα-treated HAECs and the stability of TNFα-mediated GM1 induction, an in vitro model of homeostasis upon inflammatory stimulation was designed [21]. The HAECs were treated with 1 ng/mL TNFα for 3 or 7 days and TNFα was then removed. In both experimental conditions, GM1 expression and insulin signaling were restored after removal of TNFα. On the other hand, short-term (3 days) 10 ng/mL TNFα-treated HAECs showed stable expression of GM1 even after removal of TNFα. In these GM1-stable HAECs, insulin signaling was impaired. Thus, short-term exposure to high concentrations (10 ng/mL) of TNFα can induce irreversible GM1 expression, probably resulting in chronic insulin resistance.

### 4.4. Effect of Aging/Senescence on ECs in Acute Inflammatory Vascular Models

In humans, aging/senescence affects cellular responses, leading to pathological disorders [50]. It is known that aging/senescence influences cellular signaling at the molecular level. For example, in aged fibroblasts, transforming growth factor beta 1 (TGF-β1) signaling is attenuated by reduced localization of CD44 in lipid rafts after TGF-β1 stimulation [51]. To clarify the effect of aging/senescence on the response of ECs to TNFα *in vivo*, it was examined whether short-term (3 days) exposure to 1 ng/mL TNFα could reduce eNOS levels in aged/senescent HAECs with increased GM1 expression. Short-term treatment with 1 ng/mL TNFα reduced mRNA and protein levels of eNOS in aged/senescent HAECs [22]. eNOS expression upon highly concentrated (above 10 ng/mL) TNFα exposure is regulated through NF-κB, eNOS 3′-UTR binding proteins, and miR-155 [52,53]. In aged/senescent HAECs, increased GM1 levels lead to a reduction in eNOS levels, presumably driving changes in the intensity of TNFα signaling. It is controversially known that the TNF receptor is functional on non-raft or lipid raft regions [54,55]. Increased GM1 levels reduce membrane fluidity and affect cell surface expression of raft-associated proteins [13,14,15]. Thus, it is possible that increased GM1 levels affect the cell surface distribution of the TNF receptor, leading to changes in signal transduction. It can be speculated that downregulation of eNOS levels in aged/senescent HAECs occurs via activation of eNOS 3′-UTR binding proteins and miR-155 depending on the intensity of TNFα/NF-κB signaling. Thus, even low concentrations of circulating TNFα might represent a risk factor for vascular insulin resistance in elderly people.

## 5. Future Directions and Conclusions

**Figure 2:**
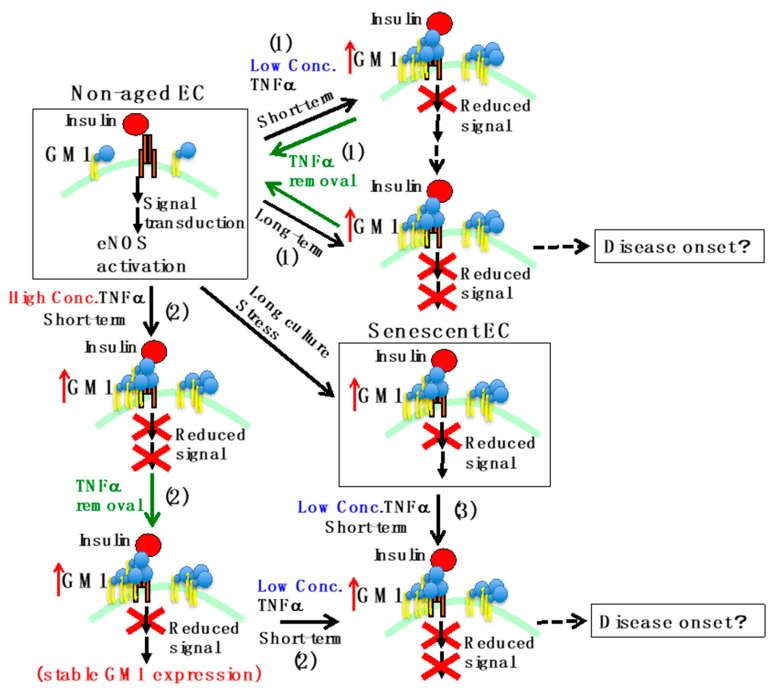
Schematic representation of insulin resistance in ECs. In non-aged ECs, activation of eNOS occurs by insulin stimulation. On the other hand, in senescent ECs, which underwent multiple cell divisions or oxidative stress, GM1 levels increase and insulin signaling decreases. The effects of TNFα are the following. 1) When short-term stimulation (acute inflammation) with low concentrations of TNFα is performed on non-aged ECs, insulin signaling decreases and GM1 levels increase. Further stimulation for a long period of time (chronic inflammation) leads to decreased eNOS expression. Removing the stimulus restores physiological conditions. 2) Short-term stimulation in non-aged ECs with high concentrations of TNFα leads to a decrease in the expression of eNOS. Even if the stimulus is removed, the increase in GM1 levels and the decrease in insulin signaling are stable. When GM1 levels are increased, short-term stimulation with low concentrations of TNFα results in decreased expression of eNOS. 3) In senescent ECs, the expression of eNOS decreases even upon short-term stimulation with low concentrations of TNFα, because GM1 levels are already increased. A continued decline in eNOS levels may lead to disease onset due to chronic reduction of NO production.

In this review, we described the molecular mechanisms of vascular insulin resistance mediated by gangliosides in aging/senescence and inflammatory conditions. As in vitro models of aging/senescence in vascular cells, ECs after multiple passages or stress-induced senescent ECs and ECs derived from subjects of different ages were used. In these ECs, the levels of GM1 increased with aging/senescence, resulting in induction of insulin resistance (Figure 2) [20]. As in vitro models of acute or chronic inflammation, the short-term (acute, 3 days) or long-term (chronic, 7 days) effect of different concentrations of TNFα on ECs was examined (Figure 2) [21]. The GM1 levels on cell membranes changed depending on time of exposure to TNFα and its concentration, and GM1 expression was found to be associated with extracellular/intracellular regulation of the insulin signaling cascade. Furthermore, aging/senescence affected the regulation of insulin resistance. Therefore, we propose that if in vitro studies assuming in vivo aging/senescence and inflammation are applied to cellular studies from other tissues and organs, there is the possibility of finding the key to solving the complexity of in vivo entities.

**Figure 3:**
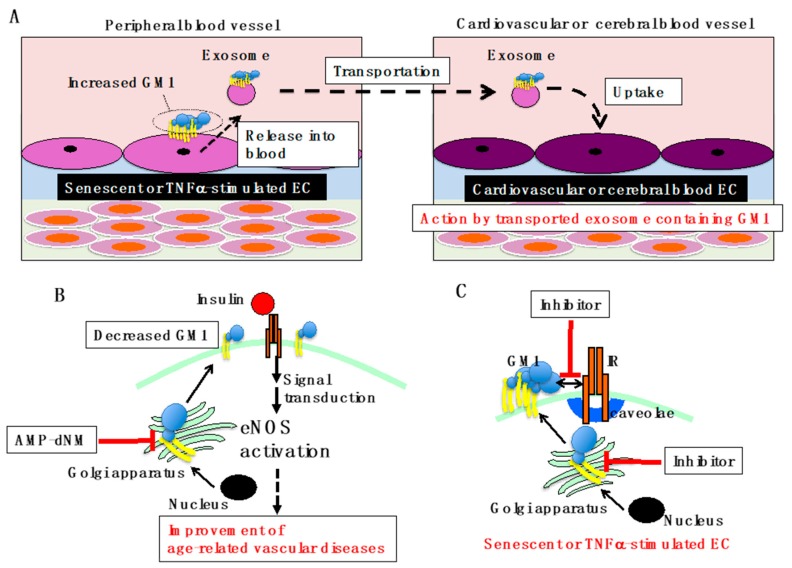
Overview of GM1-mediated vascular insulin resistance. (**A**) Proposed model for GM1-mediated vascular diseases. GM1, which is increased in ECs of peripheral blood vessels due to aging/senescence and inflammation, is secreted into blood vessels in exosomes. Exosomes containing GM1 are transported through blood vessels and incorporated by cardiovascular and cerebrovascular ECs. Vascular insulin resistance can be caused by GM1 taken up from the exosome, leading to insulin resistance-related diseases. (**B**) Improvement of age-related vascular diseases by AMP-dNM treatment. When AMP-dNM is administered, the GM1 increase in ECs associated with aging/senescence and inflammation can be suppressed. Most likely, insulin signaling can be restored, improving age-related vascular diseases. (**C**) GM1 specific inhibition for improvement of vascular insulin resistance. Specific inhibition of GM1 synthesis targeting B4GALNT1 or inhibition of the interaction between GM1 and IR in senescent and TNFα-stimulated ECs could be employed to develop new efficient therapeutic strategies for vascular insulin resistance.

Vascular insulin resistance is known to promote both atherogenesis and advanced plaque progression, leading to cardiovascular and cerebrovascular diseases [6]. Vascular insulin resistance also induces diabetes by impairing insulin-induced capillary recruitment and insulin delivery in skeletal muscle [7], and glucose-induced insulin secretion from β-cells [56]. Furthermore, brain insulin resistance caused presumably by vascular insulin resistance is considered to cause Alzheimer’s disease [57]. We showed that GM1 is involved in vascular insulin resistance in aging/senescence and inflammation at the in vitro cellular level, and we propose that GM1 is involved in the onset of vascular-related diseases. It is known that exosomes containing GM1 can be taken up by other cells and that GM1 is active in these cells [58]. Since exosomes derived from ECs can be transported throughout the whole body through blood vessels, it is possible that exosomes derived from aging or inflammation-stimulated ECs may act in cardiovascular and cerebral vessels, causing insulin resistance and leading to pathogenesis (Figure 3A).

For study of the functional roles of gangliosides, *N*-(5′-adamantane-1′-yl-methoxy)-pentyl-1-deoxynojirimycin (AMP-dNM), which specifically inhibits glucosylceramide synthase, can be used without affecting ceramide levels [59]. A previous report has shown that treatment with AMP-dNM leads to improvement of glucose tolerance, reduction of hepatic steatosis, and enhanced response to insulin in rodent models of type 2 diabetes [60,61]. Furthermore, AMP-dNM treatment was shown to reduce the development of atherosclerosis in APOE*3-Leiden and low-density lipoprotein receptor -/- mice by lowering plasma cholesterol levels [62]. Thus, gangliosides also play important roles in insulin resistance and related diseases *in vivo*. It is likely that AMP-dNM treatment, which improves insulin resistance in ECs *in vitro*, may improve insulin resistance in blood vessels in humans and may suppress age-related vascular diseases (Figure 3B).

The levels of gangliosides associated with insulin resistance differ among cell types and tissues. In adipocytes, GM3 contributes to insulin resistance in pathological conditions such as obesity [16]. In ECs, we have demonstrated that increased levels of GM1 result in insulin resistance. Experiments using transgenic mice have shown that high levels of GM1 and GM2 in muscles [17] and GM3 in the liver [18] contribute to insulin resistance. However, the key ganglioside initiating insulin resistance-related diseases in older people still has to be determined. The effect of aging/senescence on the classic targets of insulin mediated by gangliosides is also still unclear. Clarifying the effect of each ganglioside in relation to tissue-specific insulin resistance could lead to a deeper understanding of several pathological conditions, and thus to more efficient drug discovery for the treatment of insulin resistance-related diseases in elderly people. In vascular insulin resistance, as described above, GM1 is a key contributor. AMP-dNM treatment may be useful for the inhibition of vascular insulin resistance, but AMP-dNM is not a specific inhibitor of GM1 synthesis. Specific inhibition of GM1 synthesis by targeting B4GALNT1 or inhibition of the interaction between GM1 and IR could be employed to develop new efficient therapeutic strategies for vascular insulin resistance (Figure 3C).
